# Trauma-related preventable death; data analysis and panel review at a level 1 trauma centre in Amsterdam, the Netherlands

**DOI:** 10.1007/s00068-024-02576-x

**Published:** 2024-07-25

**Authors:** S. Mikdad, N. A.G. Hakkenbrak, W. P. Zuidema, U. J.L. Reijnders, R. J. de Wit, E. H. Jansen, L. A. Schwarte, J. W. Schouten, F. W. Bloemers, G. F. Giannakopoulos, J. A. Halm

**Affiliations:** 1 Trauma Unit, Department of Surgery, Amsterdam University Medical Centre, Amsterdam, the Netherlands; 2 Trauma Unit, Department of Surgery, Northwest Clinics, Alkmaar, The Netherlands; 3 Department of Forensic Medicine, Public Health Service of Amsterdam, Amsterdam, The Netherlands; 4 Trauma Unit, Department of Surgery, Medisch Spectrum Twente, Enschede, The Netherlands; 5 Department of Emergency Medicine, Erasmus MC, Rotterdam, The Netherlands; 6 Department of Anesthesiology, Amsterdam University Medical Centre, Amsterdam, The Netherlands; 7 Department of Neurosurgery, Erasmus MC, Rotterdam, The Netherlands

**Keywords:** Trauma, Morbidity, Mortality, Preventability, Quality improvement

## Abstract

**Purpose:**

Trauma-related death is used as a parameter to evaluate the quality of trauma care and identify cases in which mortality could have been prevented under optimal trauma care conditions. The aim of this study was to identify trauma-related preventable death (TRPD) within our institute by an external expert panel and to evaluate inter-panel reliability.

**Methods:**

Trauma-related deaths between the 1st of January 2020 and the 1st of February 2022 at the Amsterdam University Medical Centre were identified. The severely injured patients (injury severity score ≥ 16) were enrolled for preventability analysis by an external multidisciplinary panel, consisting of a trauma surgeon, anaesthesiologist, emergency physician, neurosurgeon, and forensic physician. Case descriptions were provided, and panellists were asked to classify deaths as non-preventable, potentially preventable, and preventable. Agreements between the five observers were assessed by Fleiss kappa statistics.

**Results:**

In total 95 trauma-related deaths were identified. Of which 36 fatalities were included for analysis, the mean age was 55.3 years (± 24.5), 69.4% were male and 88.9% suffered blunt trauma. The mean injury severity score was 35.3 (± 15.3). Interobserver agreement within the external panel was moderate for survivability (Fleiss kappa 0.474) but low for categorical preventable death classification (Fleiss kappa 0.298). Most of the disagreements were between non-preventable or potentially preventable with care that could have been improved.

**Conclusion:**

Multidisciplinary panel review has a moderate inter-observer agreement regarding survivability and low agreement regarding categorical preventable death classification. A valid definition and classification of TRPD is required to improve inter-observer agreement and quality of trauma care.

**Supplementary Information:**

The online version contains supplementary material available at 10.1007/s00068-024-02576-x.

## Introduction

Trauma-related death is used to evaluate mortality and the quality of trauma care, as trauma remains the leading cause of death among people under the age of 45 [1]. In the 1970s trauma-related preventable death (TRPD) was introduced to refer to the cases in which mortality might have been preventable under optimal trauma care conditions. This became a parameter to evaluate the quality of trauma care. TRPD was divided into three categories by Mackenzie and Shackford in the 1990s: non-preventable (TRNPD), potentially preventable (TRPPD), and definitely preventable (TRPD) [2, 3]. Hereafter, TRPD panel review has been embedded, amongst others, in the evaluation of combat casualty care [4]. This resulted in the implementation of several important innovations such as tactical combat casualty care with trauma registry data feedback, improving outcomes and reducing casualties [4].

Nevertheless, the definition of TRPD has not been unambiguous in the literature, due to differences in expert opinion on the severity of injuries and survivability. A trauma prediction algorithm was added to objectify the severity of the injury and probability of survival. However, discussion about the most suitable algorithm remains. Recently, a systematic review on the definition of TRPD was performed to identify the different definitions [5]. In conclusion, the review suggests the use of a clinical definition and a trauma prediction algorithm such as the trauma and injury severity score (TRISS) [5]. In addition, the clinical definition was based on panel review or expert opinion as presented below [5]:

TRNPD: death was unavoidable as a result of anatomic injuries, despite adequate care and appropriate evaluation and management, or due to co-morbid factors that were major contributors to death.

TRPPD: death could have been avoided, anatomic injuries that were severe but survivable under optimal care conditions; system generally appropriate, timely care, evaluation, and management generally appropriate or some deviations from standard care that could have led directly or indirectly to the death.

TRPD: death could have been avoided by the timely implementation of standard practice or was caused directly by an avoidable error, suboptimal care.

The multidisciplinary panel should include a trauma surgeon, anaesthesiologist, emergency physician, neurosurgeon, and forensic physician [5].

The aim of this study was to perform an analysis of mortality on TRPD in the region of Amsterdam, the Netherlands, and evaluate the difference in judgement by expert panel on preventability.

## Methods

All trauma patients deceased after initial presentation to the trauma bay, over the age of 18 years, between 1-1-2020 and 31-1-2022 were included. This study did not include patients whose resuscitation was discontinued before arrival at the trauma bay and who were declared dead on arrival (DOA). Additionally, the population was divided into two categories based on the Injury Severity Score (ISS); <16 and ≥ 16 (severely injured).

Patients with an ISS of 16 or higher were included and individually peer-reviewed by a multidisciplinary external review panel on preventable death. The panel composition was based on the previously published systematic review [5]. The external review panel consisted of a trauma surgeon, an anaesthesiologist, an emergency physician, a neurosurgeon, and a forensic physician. The panellists work at three different Level 1 trauma centres in the Netherlands, Amsterdam, Rotterdam and Twente, respectively. None of the panellists were involved in the presented cases. Individual experience of the expert panel ranged between 14 and 33 years. Participation of the external panel was not financially compensated and no direct relationship to the author’s institution or cases was linked. The case reports were sent by e-mail and the panellists were not informed about the identity of the other participating panellists.

Data from records of prehospital-, emergency- and in-hospital departments (i.e. radiology and operation) were collected from the electronic patient registration system and subsequently anonymized by two research fellows. Case descriptions were compiled, containing data on prehospital assessment (i.e. mechanism of injury, vital signs, treatment, and interventions), primary and secondary survey findings (i.e. additional imaging and laboratory results) and in-hospital course summarization (i.e. interventions, treatment, and complications). Five cases were chosen at random and were assessed on readability by two trauma surgeons (JH and GG). Their individual assessments were collected by the first authors (NH, SM).

A scoring form was provided to assess the cases on three items: (1) Severity of the injury (non-survivable or survivable based on the expert opinion of the panellists), (2) Errors in care (timely care, system, judgement and treatment) [6], and (3) category of trauma-related death (preventable, potentially preventable, non-preventable and preventable but with care that could have been improved (Supplementary 1)). The outcomes reported by the panellists are expert opinion. The score form was designed in an attempt to form objective parameters based on the expert opinions.

To compare the external review and outcomes of the internal review, kappa statistics were used, as well as Fleiss Kappa for the interobserver agreement between the individual panel members [5]. Kappa values of < 0 resemble poor agreement, 0.01–0.20 slight agreement, 0.21–0.40 fair agreement, 0.41–0.60 moderate agreement, 0.61–0.8 substantial agreement, and 0.81-1.00 almost perfect agreement. Categorical variables were described as percentages; continuous variables as means with standard deviation (SD). Analyses were performed using STATA for Mac, version 17.1.

## Results

A total of 95 trauma patients died after arrival at the trauma bay. In total, 36 severely injured patients (ISS ≥ 16) were selected for peer review. Overall, the mean age was 55.3 years (± 24.5), 69.4% were male and 88.9% sustained blunt trauma. The mean injury severity score (ISS) was 35.3 (± 15.3) (Table 1). Most injuries with an abbreviated injury scale (AIS) ranging from serious up to untreatable injuries (AIS 3–6) were to the head (58.3%) and chest (16.7%) (Table 1B). The median time from hospital arrival until death was 22.5 h (5.5–179.5), and 63.9% died within the first 72 h (Table 1).

**Table 1:** Demographics, traumatic fatalities ISS ≥ 16

	ISS ≥ 16 (*N* = 36)
Age, mean (SD)	55,3 (± 24.5) years
Male, N (%)	25 (69.4)
Daytime, N (%)	13 (36.1)
Anticoagulation, N (%)	7 (19.4)
DOAC	3 (8.3)
TAR	3 (8.3)
Vitamin K	1 (2.8)
ED-disposition N (%)	
Operation theatre	14 (38.9)
Intensive Care	14 (38.9)
Floor	6 (16.7)
Morgue	1 (2.8)
Coronary Care Unit	1 (2.8)
Surgery	
Surgery within 6 h, N (%)	14 (8.9)
Surgery within 24 h, N (%)	13 (36.1)
Cause of Injury, N (%)	
Accident	29 (80.6)
Assault	2 (5.6)
Self-inflicted	5 (13.9)
Mechanism of Injury, N (%)	
Blunt	32 (88.9)
Penetrating	2 (5.6)
Other	2 (5.6)
Traffic accident, N (%)	
Motor Vehicle Accident	2 (5.6)
Motor Cycle Crash	4 (11.1)
Bicycle	9 (25.0)
Pedestrian	2 (5.6)
Other	0 (0.0)
Fall, N (%)	
< 3 m	2 (5.6)
> 3 m	14 (38.9)
Unknown height	0 (0.0)
Non-accidental, N (%)	
Hit with object	0 (0.0)
Gun- and shot wound	2 (5.6)
Stab	0 (0.0)
Drowning	0 (0.0)
Other (explosion, fire)	0 (0.0)
Transport, N (%)	
HEMS	29 (80.6)
Ambulance	5 (13.9)
GCS < 9, N (%)	28 (77.8)
Prehospital intubation, N (%)	23 (63.9)
AIS > 3, N (%)	
Brain/Skull	21 (58.3)
Face	0 (0.0)
Chest	6 (16.7)
Abdomen	3 (8.3)
Spine	2 (5.6)
Pelvis	2 (5.6)
Upper extremity	0 (0.0)
Lower extremity	1 (2.8)
Soft tissue	0 (0.0)
Trauma Resuscitation Room Interventions, N (%)	
TXA	
CS immobilization	6 (16.7)
Splint	26 (72.2)
Pelvic Binder	1 (2.8)
Chest tube	6 (16.7)
Thoracotomy	7 (19.4)
CPR	3 (8.3)
	3 (8.3)
Emergency Department, Radiology, N (%)	
eFAST, positive	6 (16.7), 3 (8.3)
X-thorax	7 (19.4)
X-Pelvis	4 (11.1)
X-Spine	1 (2.8)
Total body CT-scan	34 (94.4)
Median time from hospital arrival until death (hours), median (IQR)	22.5 (5.5–179.5)
Time from hospital arrival until death, N (%)	
<1 h	2 (5.6)
2–6 h	7 (19.4)
6–12 h	3 (8.3)
12–24 h	7 (19.4)
< 48 h	2 (5.6)
< 72 h	2 (5.6)
> 72 h	13 (36.1)
Obduction, N (%)	1 (2.8)

The internal review panel judged 33 deaths as non-preventable and three as potentially preventable. The external review achieved consensus in 15 cases, and in a further eight cases, four out of five panel members reached consensus (Table 2). The inter-observer agreement between panel members is presented in Table 3. The overall kappa agreement within the external panel was moderate for survivability (Fleiss kappa 0.474) but low for categorical preventable death classification (Fleiss kappa 0.298). Most disagreements were in judging whether trauma-related death was non-preventable or potentially preventable with care that could have been improved. In total, there were 83 errors for the potentially- and non-preventable death groups. The panel judged 12 errors to have occurred in the TRNPD and 71 in the TRPPD group. Errors included system-, assessment-, treatment errors, and errors in timely care. The classification regarding the type of error is presented in Table 4. The most frequently occurring errors in care were treatment errors (i.e., resuscitation with crystalloids instead of packed red blood cells or blood components, and interventions (i.e. refrain from a chest tube thoracostomy). Examples of errors in system regard minor trauma team activation (consisting of a radiologist, nurse and surgical resident) instead of major trauma team activation (consisting of a radiologist, nurse, surgical resident, trauma surgeon, intensive care physician, anaesthesiologist and neurologist) or suboptimal prehospital triage and handover. Errors in assessment include inadequate assignment of imaging technique and errors in timely care report upon unwanted delay until assessment and further treatment.

**Table 2:**
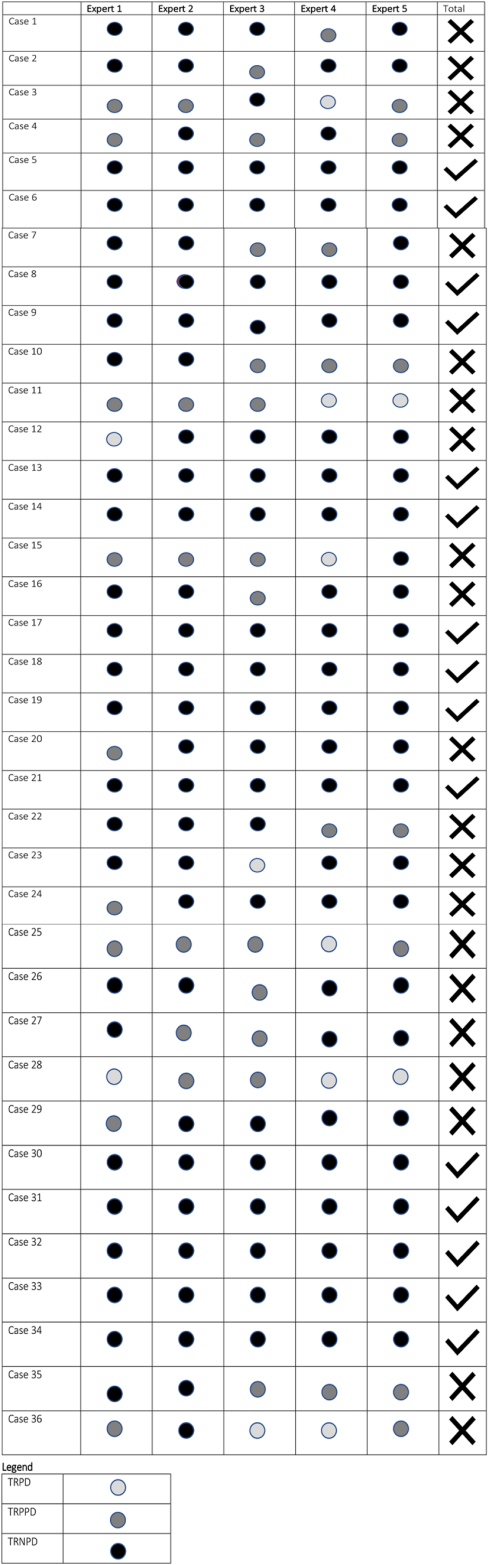
Preventability agreement between external reviewers per case

**Table 3:** Preventability agreement between external reviewers (Kappa) [4]

	Expert 1	Expert 2	Expert 3	Expert 4	Expert 5	Internal Panel
Panel 1	-	0.415	0.148	0.121	0.415	0.270
Panel 2	0.415	-	0.362	0.163	0.270	0.625
Panel 3	0.148	0.362	-	0.306	0.296	0.124
Panel 4	0.121	0.163	0.306	-	0.561	0.131
Panel 5	0.415	0.270	0.296	0.561	-	0.250

Below 0.0– Poor; 0.00–0.20– Slight; 0.21–0.40– Fair; 0.41–0.60 – Moderate; 0.61–0.80– Substantial; 0.81–1.00– Almost perfect

**Table 4:** Classification of errors in care: (A) Reported errors in care per expert. (B) Reported errors in care per category of TRPD

	Expert 1	Expert 2	Expert 3	Expert 4	Expert 5	Total
**System error**	2	1	1	0	5	9
**Error in timely care**	6	2	2	1	9	20
**Assessment error**	2	2	0	4	8	16
**Treatment error**	10	3	1	9	15	38
**Total number of reported errors per expert**	20	8	4	14	37	83
**Total number of cases with reported errors per expert**	11	5	3	10	21	50

	TRPD		TRPPD		TRNPD	
**System error**	0		1		8	
**Error in timely care**	0		2		18	
**Assessment error**	0		2		14	
**Treatment error**	0		7		31	
Total	0		12		71	

(TRPD: trauma-related preventable death, TRPPD: trauma-related potentially preventable death, TRNPD: trauma-related non preventable death)

A subgroup analysis among the acute care physicians was performed, including the trauma surgeon, neurosurgeon, anaesthesiologist, and emergency physician. Survivability was moderate (Fleiss Kappa 0.498) and agreement was low for categorical preventable death classification.

## Discussion

Trauma-related preventable death (TRPD) is one of the keystones in the evaluation of the quality of trauma care. Previous studies have shown a high variety of inter-observer agreement between panels; from disagreement up to almost perfect agreement [3, 7–9]. In order to increase inter-observer agreement in panel review a validated definition of TRPD is required. This study assessed the inter-observer (dis)agreement within a multidisciplinary panel and supports the necessity of a well-described, validated definition of trauma-related preventable death [3].

Agreement on survivability was moderate within the external panel. This is in line with previous literature on inter-observer agreement in panel review [3, 10, 11]. Unfortunately, the agreement on categorical preventable death classification was low, in 42% of the cases all 5 jury members agreed. It is noteworthy that consensus was reached only in cases judged as non-preventable death. In an additional eight cases, four out of five panel members reached consensus, the majority of the disagreements were due to differences in judgment on TRPD and TRPPD. As patient viability is assessed on different aspects by physicians (e.g., based on vital parameters, additional imaging, and response to resuscitation), this might be explained by the multidisciplinary character of the composed expert panel. Nonetheless, current literature reports benefit of a multidisciplinary panel, such as a more complete perspective on aspects of care and data evaluation [12]. Therefore, it is important to improve interdisciplinary agreement. A carefully composed panel that is willing, committed, and available to attend panel reviews can achieve high levels of agreement [12]. To improve agreement on a categorical preventable death classification, it is suggested to perform a Delphi study to reach consensus on the definition of TRPD. This is an important step in order to establish a widely supported and usable classification of preventable death.

Preventable death panel review is important to assess errors in care, thereby, increasing transparency and improving quality in trauma care. In this study, the most frequently scored errors were errors in treatment (46%) and timely care (24%). The majority of the errors (86%) occurred in the TRPPD group. Due to the severity and complexity of the injuries, it is difficult to evaluate the errors in care with regard to the injured body region. Errors in treatment were most often due to suboptimal resuscitation, i.e. crystalloids instead of blood products, small volume, and inadequate ventilation.

Despite the improvement of process-related outcomes through the implementation of an in-house attending trauma surgeon and CT scanner in the trauma bay, errors in timely care, assessment, and treatment are not uncommon. This is most likely due to the severity of the patient’s injuries [13, 14]. Moreover, by identifying errors in care, by (external) panel review, awareness is raised for the process-related outcomes and identification of the impact of the scored errors on quality of care and their relation to mortality.

In addition, the trauma severity prediction algorithm used in this study is the injury severity score. Even though, the ISS is a valid trauma algorithm used worldwide, it lacks clinically relevant outcomes [15, 16]. Prehospital time until medical care and treatment, and time until arrival at the hospital are also of great importance [10, 17]. Therefore, it may be suggested that for the assessment of trauma severity, delay in timely care and treatment should be included. It may also be suggested to include patient characteristics such as vulnerability due to age (fragility), comorbidities and medication [18, 19].

Some limitations should be noted. In this retrospective chart review, information on prehospital findings was missing, such as time variables. However, HEMS involvement was reported, and in case of great abnormality, key time variables were reported in the charts and included in the case descriptions. Furthermore, the study was designed to evaluate the cases on trauma-related (potentially) preventable and non-preventable death. During the panel review, the extent of the information that the various panel members inquired to properly evaluate preventability was variable. This was addressed by providing additional data upon request and highlighting the importance of the multidisciplinary approach to the panel review. The case descriptions were written in accordance with the abstract components for the summary of the preventable death panel review of the World Health Organization [12]. Nevertheless, despite all efforts, it remains difficult to perform a retrospective evaluation of a clinical situation without being present.

Another limitation regards the internal case assessment. Even though, the two internal experts have individually and separately judged the cases, bias could have potentially been introduced due to their long-term collaboration and case involvement. However, this was taken into consideration and also highlights the importance of multidisciplinary case assessments.

Furthermore, it may be suggested that agreement on categorical preventable death classification is even lower in the complete group of traumatic fatalities. This is hypothesized as a result of the cases in which consensus was reached, all were judged as non-preventable deaths. However, in the presented cases the ISS was 16 or higher, therefore it is more likely to contain non-preventable deaths compared to the group with an ISS of less than 16 [12].

This study features the importance of a multidisciplinary approach on identifying preventable death. Panel review is of great value in order to identify and reduce errors in care and improve outcomes. However, it also emphasizes the necessity of a validated definition of preventable death to increase inter-observer agreement and aim for consensus.

## Conclusion

(External) panel review is of great importance for the evaluation of TRPD and therefore, the quality of trauma care. However, the multidisciplinary panel review has a moderate inter-observer agreement with regard to survivability and low agreement regarding categorical preventable death classification. In order to improve inter-observer agreement and thus quality of trauma care, a valid definition and classification of TRPD is required.

## Electronic supplementary material

Below is the link to the electronic supplementary material.

Supplementary Material 1

## Data Availability

No datasets were generated or analysed during the current study.

## References

[CR1] 1. Eurostat.https://ec.europa.eu/eurostat/statisticsexplained/index.php/Accidents_and_injuries_statistics. Accessed June 28th, 2023.

[CR2] 2. Mackenzie EJ, Steinwachs D M, Bone LR, Fioccare DJ, Ramzy AI. Inter-rater reliability of preventable death judgments. *J Trauma - Inj Infect Crit Care*. 1992; 33(2):292–303. 10.1097/00005373-199208000-00021 1507296

[CR3] 3. Shackford SR, Hollingworth-Fridlund P, Cooper GF, Eastman AB. The effect of regionalization upon the quality of trauma care as assessed by concurrent audit before and after institution of a trauma system: A preliminary report. J Trauma. 1986; 26(9):812 − 20. 10.1097/00005373-198609000-00006 3746956

[CR4] 4. Eastridge BJ, Mabry RL, Seguin P, Cantrell J, Tops T, Uribe P, Mallett O, Zubko T, Oetjen-Gerdes L, Rasmussen TE, Butler FK, Kotwal RS, Holcomb JB, Wade C, Champion H, Lawnick M, Moores L, Blackbourne LH. Death on the battlefield (2001–2011): implications for the future of combat casualty care. J Trauma Acute Care Surg. 2012.73(:S431-7. 10.1097/TA.0b013e3182755dcc 23192066

[CR5] 5. Hakkenbrak NAG, Mikdad S, Zuidema WP, Halm JA, Schoonmade LJ, Reijnders UJL et al. Preventable death in trauma: A systematic review on definition and classification. Injury. 2021: S0020-1383(21)00645-8. 10.1016/j.injury.2021.07.040 34389167

[CR6] 6. Gruen RL, Jurkovich GJ, McIntyre LK, Foy HM, Maier RV. Patterns of errors contributing to trauma mortality: lessons learned from 2,594 deaths. Ann Surg. 2006; 244(3):371 − 80. 10.1097/01.sla.0000234655.83517.56 PMC1856538 16926563

[CR7] 7. Zapf A, Castell S, Morawietz L, Karch A. Measuring inter-rater reliability for nominal data - which coefficients and confidence intervals are appropriate? BMC Med Res Methodol. 2016; 16:93. 10.1186/s12874-016-0200-9 PMC4974794 27495131

[CR8] 8. Saltzherr T.P., Wendt K.W., Nieboer P., Nijsten M.W.N., Valk J.P., Luitse J.S.K., et. al.: Preventability of trauma deaths in a Dutch Level-1 trauma centre. Injury 2011; 42: pp. 870–873. 10.1016/j.injury.2010.04.007 20435305

[CR9] 9. Drake S.A., Wolf D.A., Meininger J.C., Cron S.G., Reynold T., Wade C.E., et. al.: Methodology to reliably measure preventable trauma death rate. Trauma Surg acute care open 2017; 2:10.1136/tsaco-2017-000106 PMC5877914 29766101

[CR10] 10. Pfeifer R., Halvachizadeh S., Schick S., Sprengel K., Jensen K.O., Teuben M., et. al.: Are Pre-hospital Trauma Deaths Preventable? A Systematic Literature Review. World J Surg 2019; 43: pp. 2438–2446. 10.1007/s00268-019-05056-1 31214829

[CR11] 11. Chiara, O., Cimbanassi, S., Pitidis, A., Vesconi S. Preventable trauma deaths: from panel review to population based studies. World J Emerg Surg. 2006; 12. 10.1186/1749-7922-1-12 PMC1475565 16759417

[CR12] 12. Guidelines for Trauma Quality Improvement Programmes World Health Organization International Society of Surgery Société Internationale de Chirurgie and International Association for Trauma Surgery and Intensive Care. 2009.

[CR13] 13. Hakkenbrak NAG, Mikdad S, van Embden D, Giannakopoulos GF, Bloemers FW, Schepers T, Halm JA. In-House Attending Trauma Surgeon Does Not Reduce Mortality in Patients Presented to a Level 1 Trauma Center. Prehosp Disaster Med. 2022; 37(3):373 − 77. 10.1017/S1049023X22000656 35470792

[CR14] 14. van der Vliet QMJ, van Maarseveen OEC, Smeeing DPJ, Houwert RM, van Wessem KJP, Simmermacher RKJ, Govaert GAM, de Jong MB, de Bruin IGJ, Leenen LPH, Hietbrink F. Severely injured patients benefit from in-house attending trauma surgeons. Injury. 2019; 50(1):20–26. 10.1016/j.injury.2018.08.006 30119939

[CR15] 15. Osler T., Rutledge R., Deis J., Bedrick E.: ICISS: An international classification of disease-based injury severity score. J Trauma 1996; 41: pp. 380–386. 10.1097/00005373-199609000-00002 8810953

[CR16] 16. Cole E., West A., Tai N., Brohi K.: Survival prediction algorithms miss significant opportunities for improvement if used for case selection in trauma quality improvement programs. Injury 2016; 47: pp. 1960–1965. 10.1016/j.injury.2016.05.042 27343135

[CR17] 17. Harmsen AM, Giannakopoulos GF, Moerbeek PR, Jansma EP, Bonjer HJ, Bloemers FW. The influence of prehospital time on trauma patient’s outcome: a systematic review. Injury. 2015; 46(4):602-9. 10.1016/j.injury.2015.01.008 25627482

[CR18] 18. Tan J.H., Mohamad Y., Imran Alwi R., Henry Tan C.L., Chairil Ariffin A., Jarmin R.: Development and validation of a new simplified anatomic trauma mortality score. Injury 2019; 50: pp. 1125–1132. 10.1016/j.injury.2019.01.027 30686543

[CR19] 19. Munter de L., Polinder S., Lansink K.W.W., Cnossen M.C., Steyerberg E.W.: Jongh de MAC. Mortality prediction models in the general trauma population: A systematic review. Injury 2017; 48: pp. 221–229. 10.1016/j.injury.2016.12.009 28011072

