# Supplementary material for: Trauma-related preventable death; data analysis and panel review at a level 1 trauma centre in Amsterdam, the Netherlands
Source: Eur J Trauma Emerg Surg. 2024 Jul 25;50(6):3153–60. doi: 10.1007/s00068-024-02576-x (PMC11666599; doi:10.1007/s00068-024-02576-x)
Supplement: Supplementary file 1 — Supplementary Material 1 [file 68_2024_2576_MOESM1_ESM.docx]

|  | |  |  | |  |  |  |  | | | |
| --- | --- | --- | --- | --- | --- | --- | --- | --- | --- | --- | --- |
|  | | | | | | | | | | | |
|  | | | **Preventable Death** | | | | | | | | |
|  | | | Trauma-related preventable death. A Level 1 trauma centre analysis in the Netherlands. | | | | | | | | |
| 1. | | | **Casus:** X | | | | | | |  | |
| 2. | | | **Severity of injury**   - Nonsurvivable - Survivable | | | | | | | | |
| 3. | | | **Errors in care:** | | | | |  |  |  | |
|  | | |  | | | | | *Yes* | | *No* | |
|  | | | System adequate | | | | |  | |  | |
|  | | | Timely care | | | | |  | |  | |
|  | | | Adequate assessment (diagnostics) | | | | |  | |  | |
|  | | | Adequate treatment (resuscitation, interventions) | | | | |  | |  | |
|  | | | *Comments:* | | | | | | | | |
| 4. | | | **Category:**   - Trauma Related Preventable Death - Trauma Related Potentially Preventable Death - Trauma Related Non-preventable Death - Trauma Related Preventable Death, but with care that could have been improved | | | | | | | | |
|  | | |  | | | | | | | | |
|  | | | *Comments:* | | | | | | | | |

**SUPPLEMENTARY 1: Example of Preventable Death Scoring Form**
